# Nutritional and Exercise Interventions in Cancer-Related Cachexia: An Extensive Narrative Review

**DOI:** 10.3390/ijerph19084604

**Published:** 2022-04-11

**Authors:** Vicente Javier Clemente-Suárez, Laura Redondo-Flórez, Alejandro Rubio-Zarapuz, Ismael Martínez-Guardado, Eduardo Navarro-Jiménez, José Francisco Tornero-Aguilera

**Affiliations:** 1 Faculty of Sports Sciences, Universidad Europea de Madrid, 28670 Madrid, Spain; lauraredondo_1@hotmail.com (L.R.-F.); alejandro.rubio.z@hotmail.com (A.R.-Z.); Josefranciso.tornero@universidadeuropea.es (J.F.T.-A.); 2 Grupo de Investigación en Cultura, Educación y Sociedad, Universidad de la Costa, Barranquilla 080002, Colombia; 3 BRABE Group, Department of Psychology, Faculty of Life and Natural Sciences, University of Nebrija, C/del Hostal, 28248 Madrid, Spain; imartinezgu@nebrija.es; 4 Facultad de Ciencias de la Salud, Universidad Simón Bolívar, Barranquilla 080002, Colombia; Enavarro27@unisimonbolivar.edu.co

**Keywords:** cancer, cachexia, nutrition, strength training, endurance training, physical activity, ergonutritional

## Abstract

One of the common traits found in cancer patients is malnutrition and cachexia, which affects between 25% to 60% of the patients, depending on the type of cancer, diagnosis, and treatment. Given the lack of current effective pharmacological solutions for low muscle mass and sarcopenia, holistic interventions are essential to patient care, as well as exercise and nutrition. Thus, the present narrative review aimed to analyze the nutritional, pharmacological, ergonutritional, and physical exercise strategies in cancer-related cachexia. The integration of multidisciplinary interventions could help to improve the final intervention in patients, improving their prognosis, quality of life, and life expectancy. To reach these aims, an extensive narrative review was conducted. The databases used were MedLine (PubMed), Cochrane (Wiley), Embase, PsychINFO, and CinAhl. Cancer-related cachexia is a complex multifactorial phenomenon in which systemic inflammation plays a key role in the development and maintenance of the symptomatology. Pharmacological interventions seem to produce a positive effect on inflammatory state and cachexia. Nutritional interventions are focused on a high-energy diet with high-density foods and the supplementation with antioxidants, while physical activity is focused on strength-based training. The implementation of multidisciplinary non-pharmacological interventions in cancer-related cachexia could be an important tool to improve traditional treatments and improve patients’ quality of life.

## 1. Introduction

In line with the World Health Organization, cancer is one of the principal causes of death around the world, accounting for 1 in 6 deaths, causing over 10 million deaths in 2020 alone. Among the most common are breast (2.26 million), lung (2.21 million), colon and rectum (1.93 million); prostate (1.41 million), skin (non-melanoma) (1.20 million), and stomach (1.09 million) cancer. However, 30–50% of cancers are preventable if risk factors are avoided and existing evidence-based prevention strategies are applied [1]. These approaches are physical exercise and proper nutrition, both key factors which are discussed in the present narrative review.

One of the common traits found in cancer patients is malnutrition, which affects between 25% to 60% of the patients, depending on the type of cancer, diagnosis, and treatment [1]. Malnutrition results from restricted food and nutrient intake or absorption and may be associated with disease-related inflammation [2]. Therefore, it is cancer itself and the treatment that greatly compromise the bioavailability of nutrients, as well as their absorption, leading to a degeneration in body composition (reduced lean mass, and increased fat mass and total body mass), in systemic inflammation (increased interleukin (IL)-6, IL-8, C-reactive protein (CRP), and tumor necrosis factor-α (TNF-α)) [3,4]. Indeed, this vicious circle means that approximately 79% [5] of the cancer-related patients suffer from cachexia, or loss of lean muscle mass, and changes in the architecture of the muscle sarcomere, both morphologically and functionally. Both low muscle mass and malnutrition are directly related to poorer health-related outcomes, such as increased mortality [6], postoperative complications [7], and increased risk of poorer treatment outcomes and treatment toxicities [8], along with a worse quality of life during and after the cancer treatment and recovery [9].

Thus, given the current lack of pharmacological solutions for low muscle mass and sarcopenia, holistic approaches to patient care are essential. Indeed, these holistic models which individualize nutrition and exercise have reported great changes in health-related factors of cancer patients [10,11]. However, it should be mentioned that the impact of combined nutrition and exercise on body composition in cancer patients is not clear yet, as recent reviews [12] and previous research works [13,14,15,16,17,18] suggest. One plausible explanation may be related to adherence to intervention programs, as intervention studies did not report adherence to dietary consultation and education sessions [18], reporting overall adherence to the prehabilitation intervention rather than specifying adherence to the exercise and nutrition components separately [12]. Thus, as the authors suggest, the lack of sufficient compliance-rate data in some of the studies reviewed makes it difficult to understand whether non-significant results were due to ineffectiveness of the interventions or lack of adherence to the intervention [12].

Regardless, both exercise and proper nutrition have been defined as contributing factors and true polypills [19] for major Western epidemiological diseases, including cancer. Thus, for systematic exploration, it is imperative to understand the role of dietary patterns and single nutrients in the management of cancer, offering evidence-based approaches for the nutrition management of cancer patients. The same applies to exercise interventions. Current oncology guidelines suggest a combination of resistance and aerobic training performed 3–5 times per week, at a moderate intensity, for 20–30 min, for all patients with cancer [20]. However, the ambiguity makes it so that, in terms of the density of the training load (frequency, volume, rest, and associated intensity), individualization, a key factor, is non-existent. In this line, recent reviews indicate that around only 12 to 50% of prostate cancer patients are currently following exercise guidelines and benefiting from the health advantages associated with exercise [21,22,23]. Understanding the characteristics of optimal exercise management may provide more informed clinical care with greater impact on quality of life.

Thus, the present narrative review aimed to analyze the nutritional, pharmacological, ergonutritional, and physical exercise strategies in cancer-related cachexia. The integration of multidisciplinary interventions could help to improve the final intervention in patients, improving their prognosis, quality of life, and life expectancy.

## 2. Materials and Methods

To reach the study aim, a consensus and critical review were conducted, analyzing primary sources, such as academic research, and secondary sources, such as databases, webpages, and bibliographic indexes, following procedures of previous critical narrative reviews [21,22,23]. This narrative review focused on terms describing muscle loss in the oncology patient, cachexia, malnutrition, and pharmacological and non-pharmacological interventions, and free text, when necessary, to explain certain points of the review. Three main research topics were combined: cachexia (cachexia OR anorexia OR anorexia OR weight loss OR appetite loss OR weight loss OR wasting syndrome OR metabolic dysfunction OR malnutrition OR energy malnutrition); cancer (neoplasia OR cancer); and cancer/cachexia AND pharmacological intervention OR non-pharmacological intervention OR exercise. The search was limited to the English language, with publication dates between 2000 and 2022, except for the classic literature (*n* = 3 publications older than 30 years). To cover the multifactorial nature of cancer, the databases used were MedLine, Cochrane, Embase, Psych-INFO, and CinAhl. The gray literature was not included.

Citations were selected by the authors, and they were included if they [1] addressed any topic related to cachexia, muscle wasting, metabolic dysfunction, malnutrition, and pharmacologic and non-pharmacologic interventions; and [2] described cancer patients (excluding disease-free survivors) or were published in a cancer journal. In addition, retrieved articles, practice guidelines, editorials, and letters were searched for additional references. We used the following exclusion criteria: (i) studies not related specifically to cancer; (ii) inappropriate topics, meaning those that are not relevant to the main aim of the present review; and (iii) PhD dissertations, conference proceedings, unpublished studies, abstracts, and books. All publications that met scientific methodological standards and had implications for any of the subsections of this review were included. Then data processing was performed by all review authors. Finally, the articles were discussed by the authors to draft the review prior to submission. Cachexia experts were invited to contribute additional articles. Information extraction was performed by the authors of the manuscript, who divided the information according to their area of expertise, thus dividing the text into different fragments that form the narrative line of the present narrative review.

## 3. Cancer Related Cachexia

Cachexia is a multifactorial syndrome characterized by its affection in different tissues, organs, and metabolic pathways. Its axis of pathological action begins with a process of systemic inflammation, which results in a progressive loss of the patient’s weight. In this loss, the main characteristic is negative body recomposition, increasing adipose tissue, and decreasing metabolically active tissue and lean muscle mass. This process can adversely affect patients during cancer treatment, reducing their tolerance and response to treatments, resulting in a loss of quality of life and increased mortality in patients with advanced cancer [24].

Clinically, cachexia is defined as a weight loss greater than 5% in the previous 6 months or corresponding to 2–5% for patients with a body mass index (BMI) ≤ 20 kg/m^2^ or with reduced muscle mass (sarcopenia) [25]. According to the authors of the recent reviews, its prevalence is estimated at 15.8/10,000 in Europe (2013), 16.5/10,000 in the United States (2014), and 13.4/10,000 in Japan [26]. Indeed, its epidemiology varies depending on the type of cancer. The prevalence is 80% in patients with pancreatic and gastric cancer; 60% in patients with colon cancer, prostate cancer, lung cancer, and non-Hodgkin’s lymphoma; and about 40% in patients with breast cancer, sarcoma, and leukemia. Moreover, it is considered to be indirectly associated with 20% of all cancer-related deaths [27].

**Figure 1:**
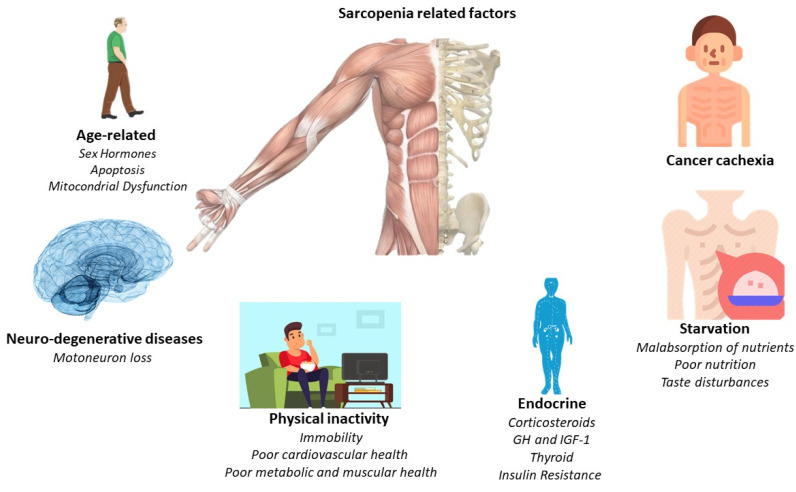
Principal sarcopenia-related factors.

The main pathological feature is the deterioration of lean muscle mass, sarcopenia. However, there are a greater number of factors which are related to the appearance of sarcopenia. For this, Figure 1 offers a quick overview of them. However, the affection is multi-organic, affecting the intestine, heart, kidneys, and liver function and morphology. With its progression, related pathologies may arise, such as cardiac arrhythmias, hypoventilation, thromboembolic events, and cardiorenal disorders [24]. Indeed, cardiac dysfunction is present in a high percentage of cancer cachexia-induced patients [28]. Thus, understanding the relationship between the heart and skeletal muscles in the clinical course of the patient with cancer cachexia is essential, linking established measures for its treatment and prevention.

### 3.1. Pathological Changes Underlying Muscle Atrophy in Cancer Cachexia

The subjacent mechanisms of cancer cachexia are multiple and intertwined. Factors related to skeletal and cardiac muscles (myokines and cardiokines) or factors secreted by cancer and cancer-associated immune cells (TGF-β, DAMPSs, and LIF) trigger a cascade of processes that ultimately result in cachexia. Circulating factors, intracellular signaling pathways, and atrophic end effectors lead to cardiac and skeletal muscle atrophy. Although the biological processes underlying cardiac and skeletal muscle atrophy are similar, their relative contribution and the specific molecular players involved differ slightly: damage-associated molecular patterns (DAMPs), growth differentiation factor 15 (GDF15), IL interleukins, leukemia inhibitory factor LIF, tumor necrosis factor alpha TNF-α, and transforming growth factor beta TGF-β [27].

### 3.2. Signaling Pathways Involved in Muscle Atrophy

**Figure 2:**
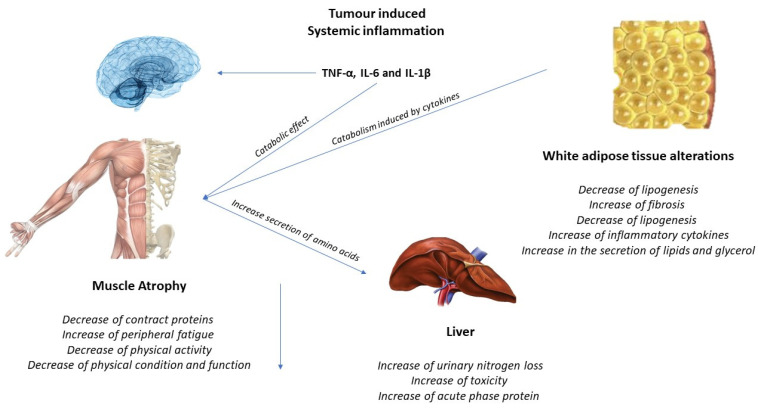
Tumor-induced systemic inflammation.

The activation of inflammatory cytokines, specifically TNF, IL-1, and IL-6, leads to activation of NF-κB and FOXO (Figure 2). Binding of IL-6 to its receptor induces STAT3 expression, leading to the activation of the NF-κB pathway. The autophagy–lysosome system is activated by the transcription factor FOXO. Activation of p38 and JAK/MAPK leads to caspase-mediated apoptosis. Myostatin also activates protein degradation through FOXO and can decrease protein synthesis by inhibiting AKT through SMAD. Levels of insulin-like growth factor-1 (IGF-1) decrease during muscle atrophy, suppressing the IGF-1 pathway, thus inhibiting protein synthesis. The ubiquitin–proteasome system (UPS) is initiated by transcription of the E3 ubiquitin ligases MuRF-1 and MAFbx/atrogin-1: ActRIIB, activin receptor type IIB; TNFα, tumor necrosis factor-α; IL, interleukin; JAK, Janus kinase; STAT, signal transducers and activators of transcription; FOXO, Forkhead box transcription factors; mTOR, mammalian target of rapamycin; NF κB, nuclear factor-κB; MAPK, mitogen-activated protein kinase; MuRF-1, muscle RING finger protein-1; IGF-1, insulin-like growth factor-1; and MAFbx, muscle atrophy F-box protein 1 [24].

### 3.3. Effects of Exercise on the Muscle and Possible Mechanisms for the Treatment of Cachexia

**Figure 3:**
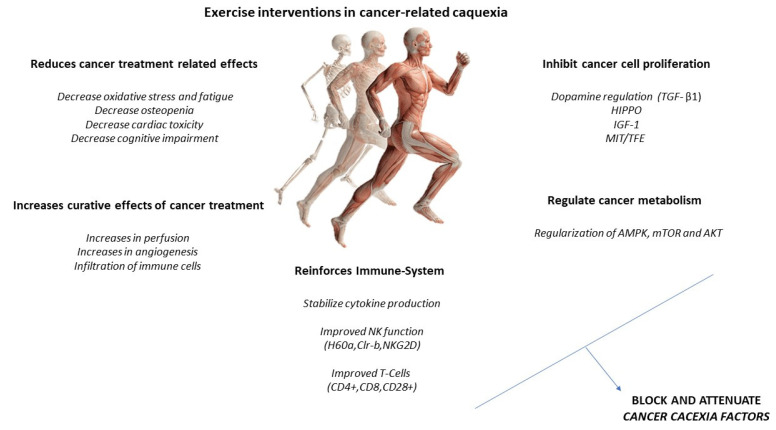
Exercise intervention in cancer-related cachexia.

Increased IL-6 may induce an anti-inflammatory effect, increasing IL-10 and IL-1ra and reducing TNF-alpha. A higher production of antioxidants than pro-oxidants may be responsible for the restoration of redox homeostasis during exercise. Increased activity of the PI3K/ALT/mTOR pathway and reduced activity of the ubiquitin proteasome and lysosomal autophagy pathways may induce protein homeostasis, increasing muscle synthesis and decreasing muscle degradation (Figure 3). PGC-1alpha activation can regulate genes involved in mitochondrial biogenesis and redox homeostasis (nuclear respiratory factors 1 and 2 and mitochondrial transcription factor A); increase GLUT-4 expression; regulate glucose metabolism; and decrease glucose metabolism, FOXO function, and proteolysis [29,30].

However, treatment and guidelines for patients with cachectic cancer have yet to be fully developed. Systemic alterations common to cancer remain viable therapeutic targets for both preventing and treating the cachectic state. Focusing on skeletal muscle maintenance has the potential to reduce systemic inflammation and improve patients’ metabolism and overall physical function. Along these lines, metabolic health and muscle mass are dramatically affected by physical activity and exercise. It is clear that physical activity and exercise are beneficial during cancer treatment and survivorship and have clear potential as a non-pharmacological treatment for muscle-wasting conditions. However, successful randomized controlled trials in patients with cachectic cancer on muscle mass, metabolism, and physical function are lacking. Further research is needed to determine the mechanistic basis for these improvements and whether these benefits can be achieved.

## 4. Cancer Related Malnutrition and Cachexia

According to the World Health Organization, “between 30–50% of cancers can currently be prevented by avoiding risk factors and implementing existing evidence-based prevention strategies” [24]. Indeed, in 2012, the American Cancer Society (ACS) Cancer Prevention Guidelines showed a strong correlation between increased adherence to dietary recommendations and decreased cancer mortality [31]. Although it is one of the protective factors, it is also a determining factor during the treatment of the oncological patient. Thus, nutritional status greatly affects outcomes in cancer patients. However, the overall incidence of malnutrition in cancer ranges from 30 to 85%, being more frequent in patients with gastric, pancreatic, lung, prostate, and colon cancer [32]. Although the etiology of malnutrition is varied, the main explanatory reason is the consequence of an imbalance between the nutritional needs of the patient, the demands of the tumor, and the availability of nutrients in the body. Thus, malnutrition is one of the explanatory factors for cachexia and weight loss. In fact, nutritional decline leading to cachexia may begin before clinical signs manifest, and most patients (about 45%) present with weight loss (10% loss) before diagnosis [33]. The implications of pretreatment malnutrition are also associated with decreased survival and increased mortality rate in patients with gastrointestinal, head, and bone-marrow-transplant cancers [34]. The implications of malnutrition on the condition, evolution, and life of the oncology patient are varied. The main one is about the patient’s life expectancy, morbidity, and mortality [25], which, either during or after treatment, are adversely affected [35,36]. There is a worse response and recovery with respect to chemotherapeutic treatment [37], with increased susceptiveness to chemotherapy toxicity [38].

A higher incidence of postoperative complications [39] and a greater deterioration and lower functionality of the immune system [40,41,42,43] were observed. Obviously, all of this translates into prolonged periods of hospitalization and rehabilitation and, therefore, higher healthcare costs. In the USA, it is estimated that the healthcare expenditure for cancer amounts to $161.2 billion, which is approximately 1.8% of its GDP [41]. Meanwhile, in the EU, healthcare spending was €57.3 billion, a total of 1.07% GDP [26].

However, when discussing malnutrition in oncology patients, we cannot use a definition as stated in the dictionary: “lack of proper nutrition, caused by not having enough to eat, not eating enough of the right things, or being unable to use the food that one does eat”. In the case of cancer patients, the definition is more complex. Both anticancer treatments and tumor and anticancer treatments are believed to result in reduced nutritional intake and metabolic abnormalities, contributing to the pathogenesis of malnutrition itself [32]. Furthermore, depending on the cancer—for example, those located in the gastrointestinal tract—patients are unable to consume sufficient nutrients, presenting reduced appetite and poor nutrient absorption [27]. Moreover, there is a process of competition between the tumor cell and the rest, competing for glucose uptake, thus fighting for its survival and nutrition [28]. Furthermore, the bioavailability and absorption of nutrients are compromised by anticancer treatments, such as chemotherapy, surgery, and local radiotherapy [29].

Indeed, the tumor also releases and induces factors related to metabolic regulation, which are addressed further in this review. The dysregulation induces changes in the energy expenditure and the metabolism of either micro- or macronutrients. Despite that there is still controversy in the literature regarding the resting energy expenditure in oncology patients, it has been proven that tumor resection normalizes resting energy expenditure in hypermetabolic patients. Thus, the elevated energy expenditure may be related to the pathogenesis itself, depending on the individual characteristics of the patient and the disease [30]. These changes in the energy expenditure will have an effect at the morphological level, and for its analysis, electrical impedance analysis (BIA) can be used. BIA is a non-invasive technique that allows for the quantification of the patient’s body composition in a short period of time. The use of this technique in parallel to the diagnosis and evolution of the patient is necessary to control not only the weight, but also the distribution of fluids, lean mass, and adipose tissue, objectifying the evolution of the patient and the effects of the nutritional intervention and physical activity [31].

The acceleration of the immune and inflammatory response is also explanatory for malnutrition processes in the patient. Cancer cells evoke a inflammatory-related immune response, which begins early in the disease and contributes to the development of malnutrition [44,45,46]. Among the inflammatory cytokines are (IL)-1, IL-6, TNF-a, and IFN-g, which are believed to promote angiogenesis and tumor growth and survival, altering nutrient metabolism, leading to malnutrition. In this line, IL-1 can also act centrally by reducing nutritional intake and leading to anorexia [47]. Within this cytokine storm, alterations at the hormonal level have also been suggested, with an increased catabolic-to-anabolic ratio; therefore, there is an organic difficulty to accumulate lean body mass, even when nutritional intake is normal [48]. In addition, tumor-derived catabolic factors have also been implicated in the pathogenesis of malnutrition, such as lipid-mobilizing factor (LMF) and proteolysis-inducing factor (PIF) or decreased leptin levels [49].

**Figure 4:**
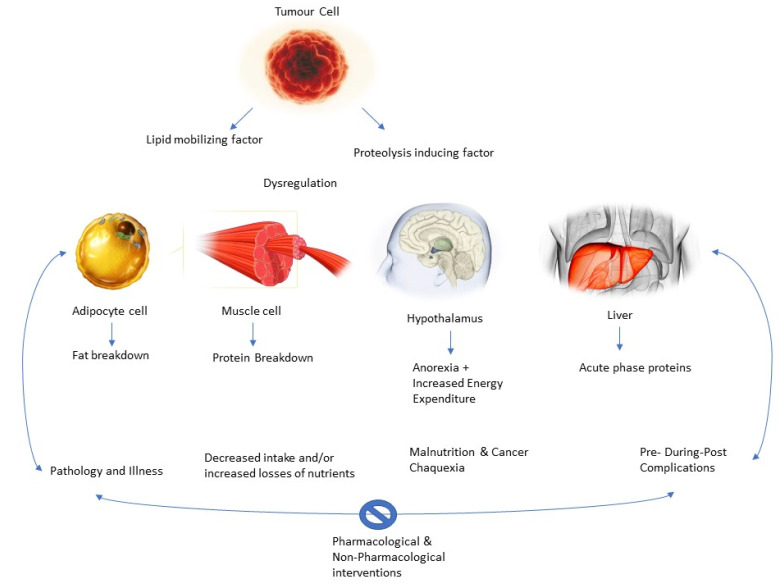
Role of pharmacological and non-pharmacological interventions.

Thus, as the authors suggest, there is a vicious circle [32]. The pathology would decrease malabsorption, the bioavailability of nutrients, and food intake, thus increasing nutrient loss and causing the patient to become susceptible to the complications mentioned above. These complications add to decreased mobility, increased fatigue, and low and poor response to the therapy, leading to a further decrease in nutritional status [29]. Thus, the cachectic state is self-perpetuating. Actual research suggests that breaking the cycle via pharmacological and non-pharmacological interventions (i.e., physical exercise and nutritional interventions) is key to the success of patient recovery and treatment (Figure 4).

## 5. Metabolic Dysregulation in the Cancer Patient

The etiology of cachexia is diverse and multifactorial, being one of the main characteristics of the loss of weight. This weight loss may be explained through two key factors: a decrease in energy intake and an augmented resting energy waste due to cancer cells’ metabolism, as tumors require the main energy shutter [33]. It has been largely described how cancer disease involves catabolic reactions to obtain energy from different sources, with the protein and lipid metabolism mainly being affected [34,35]. In fact, the principal mechanism involved in order to obtain this source of energy is muscle proteolysis, using the product of these protein catabolic reactions, degraded amino acids, the substrate used by the liver to improve gluconeogenesis [36,37]. Simultaneously, lipolysis also could be performed, resulting in free fatty acids molecules and glycerol as a product of these reactions, being gluconeogenesis substrates [37].

There are alterations in the lipid profile and metabolism, in which augmented lipolysis, decreased lipogenesis, and impaired adipogenesis and lipid deposition, as well as white adipose tissue browning, are described as factors that trigger adipose atrophy in cancer cachexia [38]. Mainly catecholamines, cortisol, natriuretic peptides, and proinflammatory cytokines are activator signals which increase lipolysis [39]. Indeed, tumoral cells are able to secrete different substances, which could modulate metabolic routes. Thus, lipolysis also could be stimulated through a tumor-secreted factor, zinc-α2-glycoprotein (ZAG), whose levels were found to be raised in cancer cachexia patients [40]. ZAG acts as a β3-adrenergic receptor agonist that activates adenylyl cyclase in a GTP-dependent process [41], ending in the result of β3-adrenergic receptor physiologic activation, triggering lipolysis and thermogenesis processes [42]. In this line, ZAG would lead to weight loss in patients with cancer, due to its capability of promoting white adipose tissue browning and energy wasting [43], increasing lipolysis. Furthermore, this metabolic dysregulation is also mediated by pro-inflammatory cytokines originated by tumor by-products, as well as by the host immune system, in which tumor necrosis factor (TNF-α), interferon-gamma (IFN-γ), and several interleukins, including IL-6, are involved [44,45]. These pro-inflammatory cytokines have been pointed out by their significant activity in weight loss by their contribution to adipose loss through two different mechanisms: by directly activating lipolysis and by reducing insulin sensitivity [46,47], causing muscle loss [45] and muscle atrophy. Moreover, an altered insulin metabolism also takes importance in the lipolysis process, since patients with cancer cachexia showed insulin resistance or reduction in its secretion [48], decreasing glucose uptake by cells, being necessary to obtain energy through the lipolysis process.

The alterations that occur at the level of protein metabolism alter the anabolic and catabolic processes, in which cachexia cancer patients regularly have either increased proteolysis or reduced protein synthesis [49]. These processes are focused into increase gluconeogenesis as a glucose energy source, as it was supported by the fact that gluconeogenesis was positively correlated to resting energy waste and negatively associated with insulin-induced protein anabolism [50]. Furthermore, muscle protein catabolism involves the activity of the ubiquitin proteasome, which includes the action of two muscle-specific E3 ubiquitin ligases, muscle ring finger 1 (MuRF1), and muscle atrophy F-box (MAFBx)/atrogin-1 [51]. These two enzymes have, in physiologic conditions, the capability of bind selective substrates and muscle proteins, such as troponin I, titin, myotilin and heavy chain myosin [52,53,54], with the aim to initiate their degradation process by the proteasome. Nevertheless, these enzymes are regulated via gene expression through different factors, including physical inactivity and malnutrition, being responsible for the increase in MuRF1 and MAFBx activity, triggering the subsequent protein degradation [55]. Additionally, pro-inflammatory cytokines also have an impact on protein catabolism, since TNF-α induces muscle wasting by MuRF1 activation [56]. Moreover, NFkB signaling secondary to pro-inflammatory cytokines triggers muscle proteolysis, including myosin heavy chain, through the activation of the ubiquitin proteasome pathway [57,58,59]. Furthermore, it has been pointed out how the muscle atrophy process is also mediated by the influence of insulin-like growth factor-1 (IGF-1), since activation of the IGF-1/phosphatidylinositol 3-kinase (PI3K)/Akt pathway may have a protective effect facing muscle atrophy by preventing the induction of these two ligases and contributing to muscle regeneration [60,61], due to the ability of IGF-1 factor stimulating myogenesis. Thus, it was also described how any substance which could impede the binding of insulin-like growth factors to the IGF-1 receptor may produce muscle atrophy [62,63].

Regarding glucose metabolism, it has been described how cachexia patients showed an increase in muscle glycolysis, resulting in a greater production of lactic acid, which is transformed into glucose through the Cory cycle [64], as well as an increase in lactic acid due to the dysfunction in energy obtention from Krebs cycle [65]. These metabolic alterations have important implications for therapeutic exercise specialists. The deeper knowledge of the metabolic and physiological implications of the cachexic oncologic patient allows the individualization of physical exercise as a therapeutic tool, already described in the scientific literature as the true polypill [19].

## 6. Pharmacological Interventions in the Cancer Patient

Pharmacological interventions for muscle loss associated with cancer treatment have been largely described in previous works in the literature. The main objective in these patients is to increase the appetite they have lost. To summarize the appetite regulation, it could be described as a complex mechanism that involves a large neural control in which the hypothalamus receives two different signals through different kinds of neuropeptides: orexigenics, such as neuropeptide Y (NPY) and agouti-related peptide (AgRP), or anorexigenics, such as proopiomelanocortin (POMC), and cocaine/amphetamine-related transcript (CART). Thus, when the hypothalamus receives these orexigenic signals in the hunger center, orexins are expressed, triggering a rising in appetite and increasing food intake. Additionally, other known appetite-stimulating substances, such as ghrelin, lead to rising NPY and AgRP expression, consequently producing the process mentioned before. Nevertheless, there are also appetite inhibitors, such as glucagon-like peptide (GLP-1) or insulin, which produce the opposite effect, decreasing NPY and AgRP expression, resulting in a decrease in appetite and food intake [66].

To increase appetite and prevent muscle mass loss, orexigens drugs were described as a useful tool which would enhance weight gain. Megestrol acetate, an active derivative of progesterone, has been pointed out as one of the most used appetite stimulants. Its mechanism of action is not well-known, but it is thought that it could be due to its capability of increasing the appetite by antagonizing the metabolic effects of catabolic cytokines, as well as due to its capability of increasing lipogenesis [67,68,69], additionally showing a good safety profile. In this line, cannabinoids also have been highlighted as an alternative to increasing appetite in cancer patients, since they have the capacity to activate the cannabinoid receptors (CB1 and CB2) in CNS, which enhances food intake and restores weight loss [70,71]. Nevertheless, compared to megestrol acetate, cannabinoids showed lower activity regarding appetite increase and weight gain, as well as worse tolerability than megestrol [68]. Similar results in regard to appetite enhancement were found for corticosteroids, which are known for their potential to stimulate appetite [72], equivalent to megestrol’s one, but also showing disadvantages associated with corticoids’ toxicity, such as immunosuppression [73]. In order to avoid these toxicity disadvantages, other appetite stimulants have been described in recent the literature, such as ghreline analogs or prokinetics (Table 1). The first ones, ghreline analogs, have recently become of interest in cancer cachexia, since ghreline, the natural ligand, has been proven to possess the ability to modulate appetite and regulate energy balance by decreasing thermogenesis [74,75], as well as the promotion of adipose tissue growth, due to the stimulation of lipogenic pathways and the regulation of skeletal muscle mass through the activation of insulin-like growth factor 1 (IGF-1) [76]. Moreover, recent studies developed in cancer cachexia patients showed how anamorelin, a ghrelin analog, significantly increased lean body mass, compared to placebo [77]. Thus, ghrelin analogs could be pointed out as an interesting pharmacological strategy which may improve muscle mass and reduce tissue atrophy. Finally, prokinetics drugs, such as metoclopramide or domperidone, used in early satiety related to cancer cachexia, also have been pointed out as useful tools in muscle loss associated with cancer management [78]. Their mechanism of action is similar in both drugs, since they act as a dopamine-2 antagonist, and a 5HT4 and weak 5HT3 receptor antagonist, leading to gastric antral contraction, decreasing post prandial fundus relaxation, and improving early satiety, due to their gastric-emptying properties [79].

Additionally, it is important to consider the inflammatory effect which cancer cell s may have per se. It has been widely described how inflammatory cytokines, such as TNF-α, IL-1, and IL-6, can be produced by tumor cells, fact which may explain the link between cancer and inflammation [80]. Furthermore, these cytokine cascade also affects tumor microenvironment, triggering tumor growth [81], generating a vicious cycle in which inflammation is the key factor which should be considered. Thus, pharmacological interventions focused on modulate inflammatory response include immunomodulatory preparations, in which TNF-α inhibitors, IL-6 antagonists, IL-6R antagonists, and TNF-α and IL-6 are the therapies that are mainly used. Moreover, a significantly increase in body weight and enhancement in nutritional status have been evidenced through the use of an IL-6R antagonist, tocilizumab, as well as a decrease in inflammatory response [82]. Additionally, ALD518, a humanized IL-6 antibody used as a IL-6 antagonist, has been demonstrated during a Phase II clinical trial in non-small-cell-lung-cancer patients a deceleration in lean body mass loss [83]. In this line, corticosteroids have also been highlighted as useful inhibitors of pro-inflammatory cytokine, including TNF-α and IL-1, modulating inflammatory response and consequently improving appetite and raising food intake [84].

Relating to the metabolic dysregulation field, antidiabetics also showed benefits in muscle loss and body weight gain associated with cancer treatment. Rosiglitazone, a PPARγ (peroxisome proliferator-activated receptor) nuclear receptor selective agonist, is used as an antidiabetic due to its capacity to reduce glycemia, decreasing insulin resistance in adipose tissue, skeletal muscle, and liver. Thus, considering this increase in insulin sensitization that may improve glucose intake by skeletal muscle cells, rosiglitazone may enhance muscle loss recovery. Additionally, it has been observed in previous studies how rosiglitazone increased weight in cancer patients by reducing body wasting [85]. Additionally, a biguanide very commonly used in diabetes treatment, metformin, also showed a potential ability to reduce lipolysis, due to its ability to induce protein phosphatase 2A (PP2A), an enzyme that owns an anti-lipolytic action [86,87], which could be useful in the treatment of cachexia in cancer patients.

Regarding drugs involved in other systems, it has been pointed out that a nonspecific β-1 and β-2 adrenergic receptor blocker, acting also as a 5HT1A antagonist, espindolol. This drug may present benefits due to its ability to preserve muscle mass, showing a reduction in muscle wasting in cancer patients [88,89], as well as in rat models [90]. The benefits perceived in cancer patients regarding to muscle loss were due to espindolol mechanism of action, including a non-selective β receptor blockade, producing a reduction in catabolism, a β2 receptor partial agonism, triggering an increase in anabolism, and a central 5-HT1A antagonism, resulting in a decrease in fatigue and thermogenesis, as well as in appetite stimulation [91,92]. Espindolol also could be useful in cancer patients due to its intricate activity that reduces heart rate and heartbeat volume, and thus cardiac output and oxygen consumption, which may be helpful in the treatment of cardiovascular alterations due to chemotherapy drugs.

## 7. Nutritional Interventions in the Cancer Patient

Nutrition is considered one of the pillars of health, and good and balanced nutrition translates into a stronger immune system [93]. It is one of the fundamental pillars during the treatment, diagnosis, and evolution of the cancer patient [94], as well as a predisposing factor for the development of certain types of cancer [95,96,97,98,99]. As such, obesity, impaired glucose metabolism, low fiber intake, red-meat consumption, and Omega 3:Omega 6 ratio imbalance increase the risk of developing cancer. With abundant proportions of fruits and vegetables in the diet and the inclusion of protective elements, such as selenium, folic acid, vitamin B12, vitamin D, and antioxidants (e.g., carotenoids), the risk of cancer is reduced [100,101,102,103,104,105,106,107]—specifically a 60–70% decrease in breast, colon, and prostate cancer, as well as a 40–50% reduction in the risk of lung cancer [107].

Despite the scientific evidence, oncology patients tend to fall into malnutrition, which can limit their response to therapies [96]; the cause is complex and multifactorial, as discussed above [97]. One of the most recent pieces of misinformation that harms the scientific community is the existing argument that nutrients may feed the tumor [98,99,100]; cancer misinformation on social media may influence caregiving behaviors and decision-making [101].

Depending on the strain of cancer and its treatment causes in the body, metabolic and nutritional interventions aim at maintaining or improving food intake, skeletal muscle mass, and physical performance, looking to mitigate metabolic derangements keeping the patient from missing treatment or reducing the dosage. Nutritional guidelines by professionals are one of the main front lines of fight as a contributing factor in the treatment of cancer patients [107,108,109,110,111,112,113,114,115]. Essentially, the key points are related to finding strategies that may help to increase or at least maintain energy levels and protein intake. However, sometimes nutritional supplements are necessary, as is discussed in the next section of this review. In extreme cases where, after the use of nutritional counseling, nutrient intake remains inadequate, enteral or parenteral nutrition may be indicated, depending on gastrointestinal tract function. In general terms, in advanced cancer cases, the benefits subtracted from nutritional therapy diminish during the weeks and days immediately preceding death [98,99,102].

Additionally, there is evidence of an increase in muscle protein anabolism with an increased dietary protein intake in cancer patients [103] that could help in muscle-loss prevention and cancer outcome and places a high-protein diet as a valid dietary strategy. The optimal protein intake has not yet been formally established; however, experts’ recommendations range from 1 g/kg/day to 1.2–2 g/kg/day [104,105,106], with it probably being closer to 2 g/kg/day [107]. Special attention is given when old age, inactivity, or systemic inflammation is present, as they are known to induce decreased responsiveness of protein synthesis to anabolic stimuli [108,109]. However, the protein supply of patients with either acute or chronic renal failure should not exceed 1.2 g/kg/day [110].

The ratio of fat and carbohydrates in feeding cancer patients has not yet been established [111]; however, it has been reported by a recent study conducted in Japan [112] that the use of a long-term low-carbohydrate diet rich in animal products was associated with increased cancer risk. In patients with metabolic problems, such as insulin resistance induced by systemic inflammation [113,114,115,116,117,118,119,120,121,122], glucose uptake and oxidation of glucose by muscle cells are impaired, as opposed to a normal or even increased fat utilization, suggesting a benefit from using a higher fat-to-carbohydrate ratio, which could be extrapolated to cancer patients [111,123]. Fat has been observed to be efficiently mobilized and utilized as a fuel source in cancer patients [124,125,126,127,128]. Several authors have demonstrated very efficient mobilization and oxidation of endogenous fat in weight-stable and weight-loss cancer patients, with intakes ranging from 0.7 to 1.9 g/kg/day [129,130,131,132,133,134,135]. Further on, energy density is important for enteral feeding, and most dietetic recommendations on cancer patients revolve around high-density foods, as they benefit patients with low appetite, early satiety, and reduced bowel motility, which are not uncommon among cancer patients [102].

In all forms of malnutrition, such as the one induced by cancer, there is a risk of micronutrients deficiency, [114,115]. Thus, dietary interventions in cancer patients should aim to fulfill the WHO/FAO recommendations on micronutrient intake, whether they are oral or enteral feeding strategies [93,116]. Moreover, in cases where recommendations are not met, the use of supplementation is considered useful and safe [117,118,119,136,137,138,139].

Regarding nutritional support, as mentioned above, it should help control symptoms and increase/maintain a diet rich in energy and protein with good fluid intake based on the patient’s tolerance [140,141,142]. Nutritional counseling is based on the study of nutritional history, diagnosis, and nutritional therapy to develop a nutritional intervention based on the measurement of energy and nutrient needs; changes in food preparation or texture; nutrient content of the diet; increasing meal frequency (with more small meals); adding energy- or protein-rich additives to meals, offering supplements when necessary; and creating different interventions to promote food intake, digestion, or absorption [142,143,144].

In addition, artificial nutrition is indicated only if patients are unable to eat adequately, and then recommending enteral nutrition first and parenteral nutrition only if enteral nutrition is not sufficient [116]. Nutritional intervention has been shown to improve body weight and energy intake, but not survival [144,145]. However, in patients undergoing radiotherapy, there is good evidence that nutritional support improves intake and weight, as well as some aspects of quality of life [114,115,146,147,148,149]. 

With the evidence available now, there is no diet known to reproducibly cure cancer or prevent cancer recurrence. As such, all forms of diets not based on scientific evidence should not be used, as they could be potentially harmful; special care should be taken for any dietary change or intervention that could heighten the risk of malnutrition or worsen the already concurring one [120]. Thus, it is very important to educate patients on what nutrition can accomplish and what it is beyond nutrition [120]. A ketogenic diet has been proposed to limit tumor-cell metabolism [121,122], with some interesting findings in in vitro [123] and animal experiments [124]. However, there are no clinical trials demonstrating the benefit of a ketogenic diet in cancer patients [116]. In this regard, it has been suggested that fasting for 24–72 h before, during, and after the application of anticancer agents could increase the efficacy and tolerance of the treatment [150,151,152,153,154,155,156]; however, this approach could put patients at risk of malnutrition.

When complications appear, maintaining nutritional status can be a challenge. In patients who are unable to eat, digest, or absorb food, artificial feeding can stabilize nutritional status. More specifically, in patients with impaired oral intake or impaired transport of food in the upper gastrointestinal tract, artificial enteral nutrition can be used [157,158]. Meanwhile, in patients with severe intestinal insufficiency, nutritional status can be maintained by parenteral nutrition [125,126,127]. Sometimes parenteral nutrition can be implemented at home; however, it is not a very extended method, as it is a complex therapy with high requirements, and the patient selection process is a highly demanding task [128].

We can conclude that the nutrition of cancer patients should be based on a high-energy diet with the use of high-density foods, such as fats, to ensure high energy intake even if the quantity of food eaten is little. Moreover, the diet should ensure a high protein intake, together with an exercise intervention to prevent muscle loss or even gain muscle in some cases. In addition, this diet should maintain all nutrients and micronutrients requirements, with artificial nutrition and supplementation being implemented when necessary.

## 8. Ergonutritional Interventions in the Cancer Patient

The consumption of dietary supplements has increased globally [129], with the use of legal ergogenic aids being especially spread in an athletic environment, going from 40 to 100% of athletes independently of their sex, sport, and performance [130].

Currently there are more than 60,000 brands available to the public [131]. There is an even greater increase in supplementation usage during the COVID-19 pandemic in hopes of strengthening the immune system [132,159,160,161,162]. People who use supplements or ergogenic aids can be divided into two main groups: amateur and professional athletes, with the objective of improving recovery and performance; and patients with chronic diseases who include physical activity as part of their treatment [163,164,165,166,167]. Furthermore, the most commonly used aids are vitamin and mineral supplements [130], such as multivitamins, multiminerals, vitamin C, vitamin D, and iron [133]. Supplements and ergogenic aids can be a good help to enhance performance, compensate deficiencies, and improve the immune system, as well as aiding in general health maintenance [133]. However, they should be treated with care and only used when truly necessary and with the supervision of a health professional, as they can cause health-related problems and side effects [134].

As discussed in the previous part of this review, nutrition is considered one of the pillars of health [93]. However, sometimes it can be tricky to maintain a good nutritional status for cancer patients only with food, as sometimes they are unable to eat, digest, or absorb food [125,126,127]. When standard nutrition is not enough, supplements can be a good help to suppress malnutrition [117,118,119]. Experts’ recommendations on protein intake in cancer patients range from 1 g/kg/day to 1.2–2 g/kg/day [118,119]; however, these recommendations do not include any indication for protein quality. Indeed, in reference to the amount and type of protein, there is no clear and concise information or guidelines. A recent scoping review analyzed different intervention studies on protein supplementation and cancer patients; a common characteristic found was that studies only addressed dose/day, without specifying a time frame and type of protein. However, given the need for individualization in protein quantity and protein type, the lack of methodological specificity is understandable, as the authors suggest [120]. What authors agree on is that the nutritional status of cancer patients is poor, especially the amount and quality of protein ingested [116,120], although it is generally underestimated that most cancer patients who require nutritional interventions for a short period of time do not need any specifically formulated amino acid mixture [98]. It has been shown that, when patients need parenteral nutrition, the inclusion of branched-chain amino acids (BCAA) in the solution results in better protein accumulation and albumin synthesis compared to standard amino acid solutions [135,136]. Further on, Deutz et al. [137] reported that the oral administration of 40 g of amino acids (0.48 g/kg) enriched in leucine and Omega 3 fatty acids to advanced cancer patients not suffering from malnutrition resulted in an increase in the rate of fractional muscle protein synthesis compared to a conventional protein supplement containing 24 g of proteins. Glutamine has also been studied for its effects on tumor-growth suppression, protein-metabolism improvement, and cancer-therapy enhancement [138]. However, there is controversial evidence, as only 8 of 24 studies using oral glutamine intake and only 6 of 12 studies using parenteral glutamine have reported a clinical benefit [139,140,141,142,143,144,145,146,147,148,149,150,151,152,153,154,155,156,157,158,159,160,161,162,163,164,165,166,167,168,169,170,171,172,173]. Furthermore, β-hydroxy-β-methylbutyrate (HMB) has been linked to the treatment of sarcopenia. Studies showed increases in muscle mass and function, as well as increases in the physical performance of older patients with or without resistance exercise. However, more studies are still needed to obtain irrefutable evidence of these effects [139].

As complications arise in cancer patients, it is hard to maintain an adequate intake of vitamins and minerals; that is why the American Cancer Society stated that the intake of multivitamin and multimineral supplements in appropriate doses (close to the recommended daily allowance) is a useful and safe strategy for cancer patients [117,118,119]. Furthermore, vitamins and trace elements supplementation is mandatory after 1 week of parenteral feeding, ensuring at least the same reference values as in oral feeding, unless the clinical situation of the patient indicates otherwise [117,140]. Specifically, vitamin D deficiency is observed in cancer patients and has been associated with the incidence and prognosis of this disease [174,175,176,177,178,179,180]. However, it is still unclear if the use of vitamin D supplementation may normalize vitamin D levels, thus improving the prognosis and having a positive effect on mortality in cancer patients [116,181].

Further on, the use of single high doses of micronutrients vastly above normal intakes is not recommended [141]. It is estimated that 50% of cancer patients consume complementary or alternative medical products, mainly multivitamin supplements [142]. Similarly, a large meta-analysis of 68 randomized trials with a total sample of over 230,000 participants found no protective effects of antioxidants, but slightly higher mortality in subjects consuming β-carotene, vitamin A, or vitamin E [143]. Similarly, a prospective observational study conducted in 290,000 men showed increased mortality from prostate carcinoma when consuming multivitamin supplements [144]. We should emphasize that these harmful effects are derived from super doses, and the use of standardized vitamin and mineral supplementation with the supervision of a health professional is safe and useful in cancer patients, as previously stated. Furthermore, high doses of micronutrients are not only detrimental to cancer patients, by they also negatively affect healthy subjects by preventing the beneficial effects of physical exercise in humans, as free radicals act as signaling molecules for anabolic processes, and an excess of antioxidants could impair these signaling pathways [145]. For example, in a randomized controlled trial, researchers found an impairment in adaptations to physical exercise when healthy subjects were supplied with vitamin C (1000 mg/day) and vitamin E (400 IU/day) for 4 weeks [146]. This detrimental effect of high doses of antioxidants could be extrapolated to physical activity interventions in cancer patients preventing the beneficial effect physical activity has in these patients. This is discussed in a posterior segment of this review. Moreover, a chronic use of antioxidants has been shown to have no beneficial effect in smokers using β-carotene (25 mg) or tocopherol (50 mg) for 5–8 years, while actually having a higher risk of developing lung cancer among those who were taking supplementation [147]. Moreover, long-term supplementation with vitamin E (400 IU/day) or selenium (200 μg) had no beneficial effect on prostate cancer incidence [148], and mortality was significantly increased in men with early prostate cancer who were supplementing with selenium in doses of more than 140 μg/day [149]. In this line, a vitamin E (400 IU/day) and vitamin C (500 mg/day) supplementation for an average of 10 years yielded no effects on cancer incidence [150].

We can summarize this discussion by underlining that supplements and ergogenic aids are just support elements. The main pillar of nutritional interventions is the diet, so we must ensure that cancer patients receive the optimal intake of each nutrient and micronutrient through food. Only when it is not possible for patients to reach requirements for health and treatment optimization should supplements be prescribed under the supervision of a health professional, and then use of such supplements should be backed by scientific evidence in regard to cancer patients. 

## 9. Physical Exercise Interventions in the Cancer Patient: Cardiovascular Exercise

It is well documented that physical activity, specifically aerobic exercise, leads to an increase in aerobic capacity, cardiovascular function, and metabolic regulation in humans [151]. This modality is carried out continuously with an intensity that resides between values of maximal oxygen consumption, ranging from 40% to 70%; an activity duration between 30 to 60 min; and a frequency of 3 to 5 times for a week [152]. Despite that aerobic exercise increases the same degree of skeletal muscle hypertrophy as resistance exercise [153], it has been shown that it can also produce skeletal muscle hypertrophy through different physiological events, such as reduction in catabolic mRNA expression, mitochondrial biogenesis, and increased muscle protein synthesis [154,155,156]. In this line, it could be postulated as an interesting non-pharmacological strategy in older adults and other clinical populations, such as cancer patients, who are experiencing muscle loss [157]. In this line, several studies have reported that aerobic exercise produces the activation of AKT/mTOR pathway, reducing protein degradation [158,159] and decreasing intracellular ROS production by protecting muscles through antioxidant effect [160].

According to the progression of cancer cachexia, Fearon et al. [25] reported that it should be understood as a continuous process starting with pre-cachexia, moving toward cachexia, and ending with refractory cachexia. In this line, the practice of physical exercise evokes an anti-inflammatory response, which is a direct biomarker of muscle wasting [181,182,183,184,185,186,187,188,189,190] and one of the main problems associated with cachexia. However, most studies that have implemented an aerobic-exercise intervention to observe the effect on muscle loss during cachexia have been performed in animal models. In this line, a recent meta-analysis [161] established that the main cause of this fact is the late but rapid development of cachexia in the last stages of the disease, making it difficult to carry out RT or RCT in human models. 

### 9.1. Aerobic Exercise Interventions in Animal Models

In the majority of studies using animal models, the development of cachexia was induced through tumor inoculation. In this line, Khamoui et al. [162] reported that 60 min/5 days per week in motorized wheels did not prevent weight loss but contributed to maintaining body function and recovering a percentage of muscle mass after tumor-induced cachexia in murine rodents. Moreover, Ballarò et al. [160] conducted a preclinical study in colon-carcinoma-bearing mice and informed that moderate exercise, 3-days at 11 m/min for 45 min in motorized wheels, significantly decreased body weight loss, increasing muscle mass and appetite compared to the control mice. Moreover, Gholamian et al. [163] reported that the group of female Balb/c mice that started aerobic physical exercise before breast-cancer induction and continued after it prevented muscle dysfunction and atrophy caused by tumor cachexia. Similar results were found by Shamsi et al. [164] after 12 weeks (6 weeks prior to breast cancer tumor injection and 6 weeks after it) aerobic training on a treadmill for 5 days/week in female Balb/c mice. In this line, Moreira et al. [165] reported that aerobic training performed during 8 weeks in treadmill running, 3 days/week, 44 min/day, at 55–65% VO2max, significantly decreased cachexia in adult male Wistar rats with an induced tumor. Furthermore, Alves et al. [166] showed that aerobic interval exercise, three bouts at 85% at maximal speed for 4 min, with intervals at 60% of maximal speed for 3 min, attenuated muscle loss in the plantaris muscle of rats bearing bone cancer. Despite the results, Niels et al. [161] established in a meta-analysis that, due to significant heterogeneity among designs (20 preclinical trials) and training modalities used, no real differences were observed between control and intervention (exercised mice) regarding body mass or muscle mass. Moreover, certain studies implemented training before inoculating the tumor in rodents and others after inoculation, and this may have affected the conclusions obtained.

### 9.2. Aerobic Exercise Interventions in Human Models

As mentioned above, due to the developmental characteristics of cachexia, it is difficult to establish the role of physical exercise in cancer patients. In this line, no RT or RCT has been carried out to evaluate the application of an aerobic exercise program in isolation in cancer patients with cachexia. In this sense, a recent meta-analysis [167] included only four RCTs that evaluated the effect of physical exercise on cancer cachexia. However, the authors reported that the evidence is very low, due to the low number of studies carried out, to establish an improvement in muscle mass loss after the application of an exercise program compared to traditional treatment. In addition, it seems that multimodal training (the combination of different physical training modalities as aerobic and strength exercises with medication and/or nutritional supplementation) could be a much more interesting strategy to obtain better results in this type of clinical population [168].

### 9.3. High-Intensity Interval Interventions

Despite the positive adaptations associated with aerobic-type strategies in cancer patients, high-intensity interval training emerged as a much more time-efficient strategy that is capable of promoting metabolic and brain adaptations to a greater extent than aerobic exercise [169,170]. In addition, HIIT has been shown to be a safe [171] and effective strategy to increase parameters related to cardiovascular fitness (VO2max) in cancer patients [172]. Due to these positive adaptations, it could be an effective strategy to reduce the loss of muscle mass caused by cancer [173]. In this line, Ahmadabadi et al. reported that 4 weeks of HIIT (six intervals of 3 min and 20 s at 80–95% VO2max, with one-minute recovery between each interval at 30–35% VO2max) reduced muscle wasting during tumor progression in breast-cancer-bearing mice with cachexia [174,175]. However, Alves et al. observed an improvement in running capacity and muscle contractility without changes in muscle mass after 16 days of five intervals of 3 min running at 18 m.min^1^, followed by 4 min running at 25 m.min^−1^, in mice with tumor induced [176]. In humans, Niels et al. showed a maintenance of weight and BMI and improvements in muscular strength during 7 months of high-intensity training (a combination of resistance and endurance training) in a 46-year-old male with a stage IV pancreatic cancer [177]. Although more studies are needed to assess the effect of a HIIT protocol on cachexia-induced muscle loss, current guidelines state that HIIT is safe, tolerable, and effective as part of a comprehensive cancer rehabilitation program [178].

## 10. Physical Exercise Interventions in the Cancer Patient: Strength Exercise

It is known that strength training (ST) improves strength and promotes skeletal muscle hypertrophy by progressively overloading the muscles through external loads [179]. In this line, this modality is associated with performing different exercises by using the bodyweight or other equipment developed with variable intensities commonly expressed as a percentage of a maximum repetition (RM) [152]. During cancer cachexia, signaling and metabolic pathways that increase protein synthesis are suppressed [180], leading to loss of muscle mass. However, it is well established that TS can increase protein synthesis and activate the mTORC1 pathway [191], which appears to be an important mechanism for hypertrophy and a potential strategy to mitigate cachexia-induced muscle loss [24,192]. Concretely, this kinase activates downstream substrates such as p70S6k, which is directly involved during the signaling pathway for muscle protein synthesis [181]. However, a recent review established that aerobic exercise could be more beneficial than resistance training in counteracting muscle loss during cachexia [182]. However, this conclusion is based on studies in animal models (see Section 8), due to the difficulty of finding patients who engage in long-term regular exercise during the development of the disease. In addition, aerobic training sessions may be too long to be carried out on a daily basis in conjunction with cancer treatment per se, which also consumes too much time of patients during and after treatment [183]. Thus, strength training sessions could be postulated as a good alternative to reduce the negative effects of cachexia in skeletal muscle to be able to be carried out in less time and in an indoor way. In this vein, Capozzi et al. [184] implemented a 12-week lifestyle intervention and progressive ST program in a cancer patient in radiation treatment or immediately after completion. Despite that intervention while undergoing treatment did not reduce the loss of lean body mass, the patients who carried out the training program until the end of the treatment showed greater adherence to physical exercise, a fact which may have important clinical implications.

Several studies have implemented a combination training program, such as concurrent training, in a patient with cancer. In this line, Newton et al. [185] reported that strength training on its own provided greater increases in appendicular muscle mass than strength training accompanied by more than 20–30 min of low-intensity aerobic training in prostate cancer patients. These results could be logical, taking into account the interference phenomenon that occurs when both modalities are combined [186]. However, 10 weeks of supervised aerobic and resistance training (60 min/day) in cancer patients showed an improvement and increase of antioxidant capacity and muscular strength, along with a reduction of protein markers and DNA oxidation [187]. In this line, Galvāo et al. [188] carried out a 12-week combination program of aerobic (15 to 20 min of cardiovascular exercises at 65 to 80% of patients’ max heart rate and a rated perceived exertion scale of 11 to 13) and strength (full-body workout with intensity progress from 12RM to 6RM and two to four sets per exercise). The results showed that patients undergoing exercise showed an increase in lean mass and strength level than patients treated with usual care. Moreover, 12 weeks of strength training in combination with electrical muscle stimulation performed twice a week significantly improved muscle mass and body weight in patients with advanced lung cancer [189]. In another study with 41 head-and-neck patients, the patients were divided into two experimental groups that carried out 12 weeks of strength training and 12 weeks of self-selected physical activity, but in a different order. After the intervention, both groups increased lean body mass and muscle strength at a similar level [190]. Furthermore, Donatto et al. [183] reported that mice that had a tumor induced but underwent eight sessions of resistance training exhibited a minor loss in total muscle weight and in the amount of glycogen in the gastrocnemius. These events are related to the main consequences during cachexia. Conversely, Khamoui et al. [162] established that neither mode of training (cardiovascular or strength training) was able to prevent bodyweight loss in rats. Furthermore, cardiovascular training appeared to be more beneficial than strength training, since the latter induced the expression of genes associated with muscle damage. However, Kamel et al. [191] reported improvements in muscle mass in both the upper and lower limbs of patients with pancreatic cachexia after 12 weeks of whole-body ST with an intensity of 50–80% of 1RM, three sets, and 8–12 repetitions than group control.

According to training intensity, HIIT can be a very interesting strategy to obtain positive adaptations in skeletal muscle in cancer patients. In this line, a recent meta-analysis has established that incorporating resistance exercises into this type of strategy can reduce the muscle wasting caused by cachexia by regulating the deficiencies in skeletal muscle and adipose tissue that occur during this phenomenon [192]. However, there is not much evidence of HIIT programs with resistance exercises, because this type of training has been shown to produce much lower exercise adherence rates than aerobic exercise [193]. In this line, only Cormie et al. performed a HIIT protocol with high- or low-intensity resistance exercises in women with lymphedema related to breast cancer. The training sessions for the group that trained with the highest intensity included six upper-body exercises and two lower-body exercises with high loads (75–85% of 1RM). Results showed that moderate-to-high-intensity resistance exercise significantly improves muscular strength and muscular endurance in this population than low-intensity exercise [194].

Although the results of the studies are very heterogeneous, as well as the methodologies used, it seems that strength training can increase or at least maintain the loss of muscle mass experienced during cachexia. However, it is recommended that this type of strategy be carried out in combination with other techniques (other training modality, nutrition, or supplementation control) to address it from a multimodal point of view, since cachexia comes from multiple metabolic pathways that contribute to this condition [168].

## 11. Key Points

− Malnutrition in oncological patients is a complex process of diverse etiology. A complex system of organic responses related to the patient’s systemic inflammation is responsible for producing a vicious circle.
− The pathology itself decreases food intake, leads to malabsorption, and increases nutrient loss, causing the patient to become more susceptible to complications. These complications add to the patient’s level of illness, including increased fatigue, poor response to therapy and dynapenia, adding further complications, and altering nutritional status. Thus, the cachectic and malnourished state is self-perpetuating.
− Pharmacological interventions in the treatment of cancer patients produce a negative effect on the patient’s inflammatory state. Physical exercise, nutrition, and ergogenic aids are postulated as key tools to attenuate the chronic inflammatory state.
− Adjuvant pharmacological interventions parallel to the treatment of the patient during chemotherapy and radiotherapy are essential, without neglecting the main non-pharmacological aids, nutritional and ergogenic interventions, and physical exercise.
− Co-adjuvant therapies of physical exercise and nutrition are effective in more than 26 chronic diseases, including cancer [195], with type I scientific evidence.
− Nutritional interventions aim to maintain or improve food intake, skeletal muscle mass, and physical performance, looking to mitigate metabolic derangements and keeping the patient from missing treatment or reducing the dosage. Cancer patients’ diet should be based on a high-energy diet with the use of high-density foods, such as fats, to ensure high energy intake even if the quantity of food eaten is little. Diet should maintain all nutrients and micronutrients requirements with a high-protein intake, together with an exercise intervention to prevent muscle loss or even gain muscle.
− Artificial nutrition and supplementation should be implemented when necessary if it is not possible for patients to reach requirements for health and treatment optimization. Supplements should be prescribed under the supervision of a health professional.
− Regarding protein intake, the amount and type, there is no clear and concise information or guidelines. However, the authors agree that nutritional status of cancer patients is poor, especially the amount and quality of protein ingested. A minimum range from 1 to 1.2–2 g/kg/day is suggested without specifying the type or time frame of intake.
− The ergogenic aids are focused on the implementation of antioxidant, vitamin D and anticatabolic substances as β-hydroxy-β-methylbutyrate.
− Multimodal training (the combination of different physical training modalities, such as aerobic and strength exercises, with medication and/or nutritional supplementation) could be an interesting strategy to obtain better results.
− Among all strategies, strength training has shown the greatest results for increasing or at least maintaining the loss of muscle mass experienced during cachexia. However, while adding another stressor stimuli as physical exercise, it is recommended to implement it in combination with other non-pharmacological interventions, such as nutrition or supplementation, to address it from a multimodal point of view.

In addition, as mentioned above, the treatment of adverse phenomena experienced by cancer patients could be carried out in a multidisciplinary or multimodal way, combining pharmacological and non-pharmacological strategies (exercise and nutrition) [196]. However, in patients with cachexia, there is uncertainty about the benefit of combining physical exercise and other therapies to reverse or maintain muscle loss. In this sense, a recent meta-analysis established that the combination of strength or aerobic training in combination with different nutritional supplements (omega-3 fatty acids and branched-chain amino acids) could be beneficial to treat this phenomenon [168]. In relation to the influence of this multimodal model on other parameters, Schmiit et al. conducted a 3-week multimodal rehabilitation program (exercise, medical and therapeutic treatment, and educational sessions of nutrition, among others) in women who had been treated for cancer [197]. These authors established a HIIT training group (eight 1-min sessions at >95% HRpeak of intense walking interspersed by 2-min intervals of slow walking as recovery) or low-intensity training group (six 75-min sessions of moderate intensity at 60% HRpeak). Although the results were similar with HIIT and the low-intensity group, HIIT may be a time-efficient strategy, because it takes less time to be performed. In addition, the authors state that it is difficult to establish whether the increases in certain psychophysiological aspects of health are due solely to physical exercise or to the combination of exercise and nutritional recommendations [197]. Moreover, Adamsen et al. found that a 6-week multimodal intervention (high-intensity cardiovascular and resistance training, relaxation and body awareness training, and massage) for tumor patients undergoing adjuvant chemotherapy did not improve quality of life [198]. In contrast, Midtgaard et al. showed that the 12-month multimodal program (individual and group counseling in combination with high-intensity group exercise training once a week) improved quality of life and VO2max in cancer survivors after treatment. In relation to the evidence shown, the multimodal strategy could be more attractive to be carried out by cancer patients who have adherence problems to individual physical exercise programs, as well as treating certain deficits with other methods that physical exercise cannot improve [22,199,200].

## 12. Conclusions

Cancer-related cachexia is a complex multifactorial phenomenon in which systemic inflammation plays a key role in the development and maintenance of the symptomatology. Pharmacological interventions produce a negative effect on the inflammatory state and cachexia. Nutritional interventions are focused on implementing a high-energy diet with high-density foods and supplementation with antioxidants, while physical activity is focused on strength-based training. The implementation of multidisciplinary non-pharmacological interventions in cancer-related cachexia could be an important tool to improve traditional treatments and improve patients’ quality of life.

## Figures and Tables

**Table 1:** Pharmacological interventions: activities and results.

Molecules	Activity	Results
Megestrol acetate	Stimulates appetite	Orexigens (Increase food intake and/or decrease body wasting)
Cannabinoids		
Ghreline analogs (anamorelin)		
Prokinetics (Metoclopramide, Domperidone)	Modify gastric emptying properties	
Tocilizumab	Immunomodulator	Increase in body weight Enhancement in nutritional status Decrease in inflammatory response
ALD518 (IL-6 antibody)	Immunomodulator	Deceleration in muscle loss
Rosiglitazone	Decreases insulin resistance in adipose tissue, skeletal muscle, and liver	Improves glucose intake Decrease body wasting
Metformin	Decrease lipolysis	Improves weight gain
Espindolol	Decreases catabolism Increases anabolism Decreases thermogenesis	Decreases body wasting and fatigue

## Data Availability

Not applicable.
